# *CBFB*-*MYH11* hypomethylation signature and *PBX3* differential methylation revealed by targeted bisulfite sequencing in patients with acute myeloid leukemia

**DOI:** 10.1186/s13045-014-0066-4

**Published:** 2014-09-30

**Authors:** Hana Hájková, Markus Hsi-Yang Fritz, Cedrik Haškovec, Jiří Schwarz, Cyril Šálek, Jana Marková, Zdeněk Krejčík, Michaela Dostálová Merkerová, Arnošt Kostečka, Martin Vostrý, Ota Fuchs, Kyra Michalová, Petr Cetkovský, Vladimír Beneš

**Affiliations:** Department of Molecular Genetics, Institute of Hematology and Blood Transfusion, U Nemocnice 1, Prague, Czech Republic; European Molecular Biology Laboratory (EMBL), Core Facilities and Services, Meyerhofstraße 1, Heidelberg, Germany; Clinical Department, Institute of Hematology and Blood Transfusion, U Nemocnice 1, Prague, Czech Republic; Center of Oncocytogenetics, Institute of Medical Biochemistry and Laboratory Diagnostics, General University Hospital and First Faculty of Medicine, Charles University in Prague, U Nemocnice 2, Prague, Czech Republic

**Keywords:** Acute myeloid leukemia, *CBFB*-*MYH11*, DNA methylation, Targeted bisulfite sequencing, *PBX3*

## Abstract

**Background:**

Studying DNA methylation changes in the context of structural rearrangements and point mutations as well as gene expression changes enables the identification of genes that are important for disease onset and progression in different subtypes of acute myeloid leukemia (AML) patients. The aim of this study was to identify differentially methylated genes with potential impact on AML pathogenesis based on the correlation of methylation and expression data.

**Methods:**

The primary method of studying DNA methylation changes was targeted bisulfite sequencing capturing approximately 84 megabases (Mb) of the genome in 14 diagnostic AML patients and a healthy donors’ CD34+ pool. Subsequently, selected DNA methylation changes were confirmed by 454 bisulfite pyrosequencing in a larger cohort of samples. Furthermore, we addressed gene expression by microarray profiling and correlated methylation of regions adjacent to transcription start sites with expression of corresponding genes.

**Results:**

Here, we report a novel hypomethylation pattern, specific to *CBFB*-*MYH11* fusion resulting from inv(16) rearrangement that is associated with genes previously described as upregulated in inv(16) AML. We assume that this hypomethylation and corresponding overexpresion occurs in the genes whose function is important in inv(16) leukemogenesis. Further, by comparing all targeted methylation and microarray expression data, *PBX3* differential methylation was found to correlate with its gene expression. *PBX3* has been recently shown to be a key interaction partner of HOX genes during leukemogenesis and we revealed higher incidence of relapses in *PBX3*-overexpressing patients.

**Conclusions:**

We discovered new genomic regions with aberrant DNA methylation that are associated with expression of genes involved in leukemogenesis. Our results demonstrate the potential of the targeted approach for DNA methylation studies to reveal new regulatory regions.

**Electronic supplementary material:**

The online version of this article (doi:10.1186/s13045-014-0066-4) contains supplementary material, which is available to authorized users.

## Background

Changes in DNA methylation patterns are a known hallmark of acute myeloid leukemia (AML) and underlie AML pathogenesis [1]. DNA methylation in patients with AML has been studied extensively and may reflect either specific molecular abnormalities or characterize a group of patients without an apparent molecular aberration. Specific translocations such as *PML*-*RARA*, *AML1*-*ETO* (*RUNX1*-*RUNX1T1*), *MLL* translocations or *CBFB*-*MYH11* fusion, as well as *CEBPA*, *NPM1*, *IDH1*/*IDH2, DNMT3A*, *TET2* and *RUNX1* mutations have been described to display distinct methylation signatures [2-4]. These epigenetic profiles are usually accompanied by specific gene expression features. Studying genes that are epigenetically deregulated in different groups of patients may contribute to a more detailed understanding of pathways involved in the leukemic transformation. Importantly, the effect of DNA methylation changes is greatly dependent on the location of differentially methylated regions (DMRs) [5]. New approaches using next-generation sequencing enable studying of DMRs scattered throughout the genome and targeted bisulfite sequencing offers a reasonably balanced ratio between cost and informativeness (number of CpGs covered) [6]. The link between gene expression and DNA methylation data is needed to find pathologically relevant DNA methylation changes, especially because many (or even the majority of) DMRs reflect the tissue of origin and not leukemia (cancer) specific changes [7].

In this study, 84 megabases (Mb) of 14 AML genomes and one CD34+ pool of cells from healthy donors were captured for DNA methylation and gene expression profiling. The aim was to identify differentially methylated genes with potential impact on AML pathogenesis based on the correlation of methylation and expression data.

AML patients with *CBFB*-*MYH11* fusion (*CBFB* - Core-binding factor, beta subunit; *MYH11* - Myosin, heavy chain 11) resulting from inv(16) rearrangement clustered together in a hierarchal DNA methylation and expression analysis. The majority of differentially methylated regions unique for *CBFB*-*MYH11* patients were hypomethylated and genes assigned to such regions were previously described as overexpressed in inv(16) AML [8].

*PBX3* (pre-B-cell leukemia homeobox 3), recently demonstrated as an important cofactor of *HOXA9* in leukemogenesis [9], was validated as a gene whose gene expression levels correlated with DNA methylation of its putative regulatory region across AML subtypes. The importance of *PBX3* is underlined by the fact that *PBX3*-overexpressing patients relapse more frequently. In summary, we discovered new genomic regions affected by aberrant DNA methylation that are associated with expression of genes implicated in leukemogenesis.

## Results

### Inv(16) methylation and expression cluster

We performed targeted bisulfite sequencing to discover specific methylation changes in 14 AML samples of diverse clinical and genetic background versus a pool of CD34+ healthy control cells (see Table 1 for patients’ characteristics). Agilent’s SureSelect^XT^ Human Methyl-Seq system was used to interrogate DNA methylation of selected regions (84 megabases in total) of their genomes. These targeted regions comprise CpG islands, CpG shores and shelves, as well as cancer and tissue-specific DMRs. For all samples, on-target rates were very good (about 95% for +/− 100 bp), while the percentage of targets above 20× coverage varied more widely (between ~ 70% and 90%). Targeted methylation data were correlated with previously obtained whole-genome bisulfite (WGBS) data (unpublished) for 3 cross-experiment (WGBS/target enrichment) samples. Taking into account positions with minimal coverage of 10, there was a strong positive correlation (R ≥ 0.97) in all cases.

**Table 1:** **Characteristics of AML patients**

**Sample ID**	**Genetic aberration**	**Clinical characterization**	**FAB subtype**	**Gender**	**Material**	**% blasts**
**AML_1**	*CBFB*-*MYH11*, *FLT3-*ITD	*de novo* AML	AML M4	male	BM	68
**AML_2**	*CBFB*-*MYH11*	*de novo* AML	AML M4	female	PB	40
**AML_3**	*NPM1*	therapy-related AML*	AML M1	female	PB	67
**AML_4**	*MLL*-PTD	therapy-related AML*	AML M4	female	BM	42
**AML_5**	*NPM1*, *DNMT3A*, *FLT3-*ITD	AML with multilineage dysplasia	AML M2	female	PB	58
**AML_6**	none	AML with multilineage dysplasia	AML M1	female	BM	82
**AML_7**	*NPM1*, *DNMT3A*, *FLT3-*ITD	*de novo* AML	AML M4	male	BM	94
**AML_8**	*FLT3-*ITD	*de novo* AML	AML M1	male	BM	87
**AML_9**	*FLT3-*ITD	*de novo* AML	AML M1	male	BM	89
**AML_10**	*NPM1*, *FLT3-*ITD	*de novo* AML	AML M2	female	BM	75
**AML_11**	*MLL*-PTD	*de novo* AML	AML M1	male	BM	92
**AML_12**	*MLL*-PTD, *CEBPA*	*de novo* AML	AML M1	female	PB	80
**AML_13**	*NPM1*, *CEBPA*	AML after MDS	AML M6	male	PB	60
**AML_14**	none	AML after MDS	AML M2	male	PB	44
**CD34+**		pool of 4 CD34+ healthy control cells		males		

AML_1 to AML_14 are AML patients at diagnosis that were subjected to targeted bisulfite sequencing and gene expression profiling; CD34+ is a healthy control’s pool; ^*^after breast cancer; BM – bone marrow, PB – peripheral blood.

**Figure 1:**
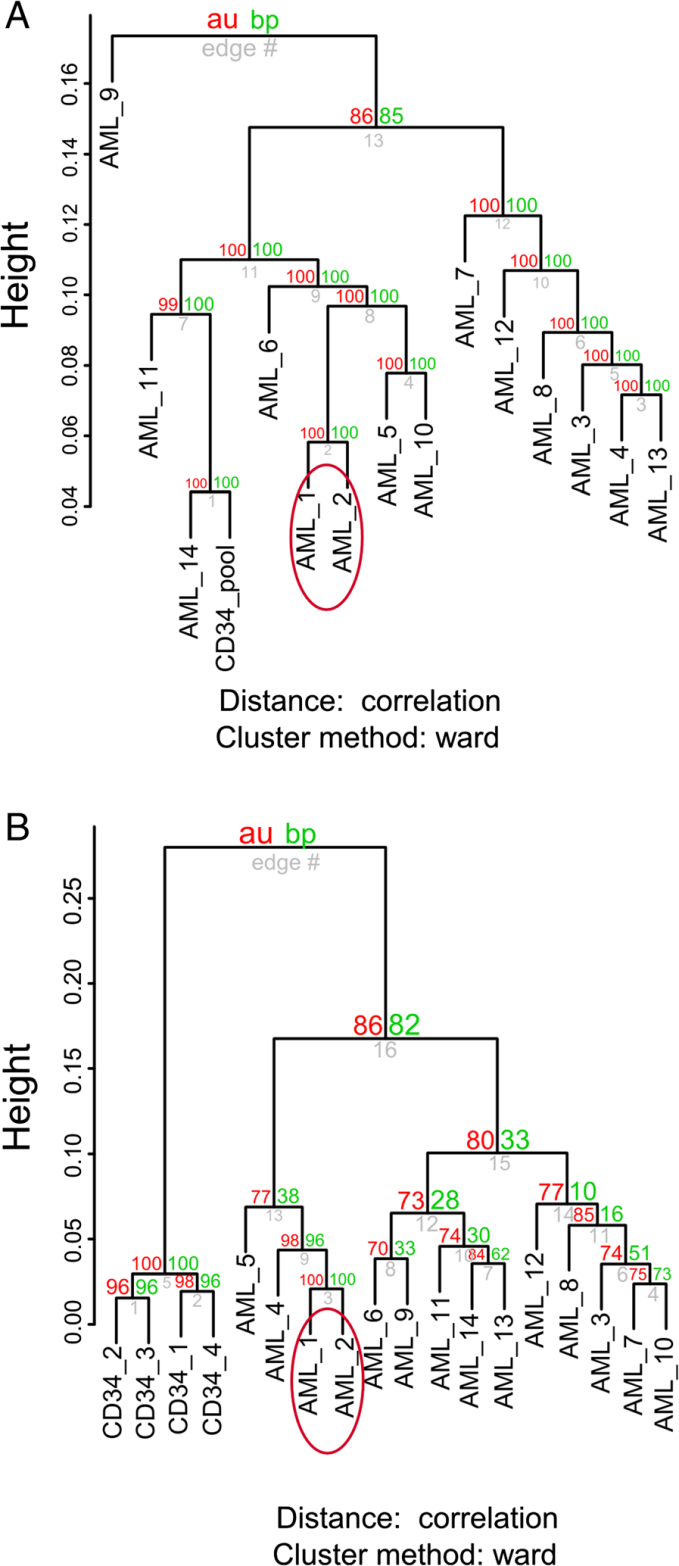
**Inv(16) methylation and expression cluster. (A)** Hierarchal DNA methylation clustering of CpGs (n = 2 010 310) using the correlation as a distance measure and Ward’s method (AML_1 to AML_14 – AML patients; CD34_pool – healthy control’s CD34+ pool) indicating *CBFB*-*MYH11* methylation cluster (in ellipse), other molecular abnormalities did not form clusters neither clinical characteristics did it; **(B)** Hierarchal gene expression clustering of altogether 8884 genes with a detection P value ≤ 0.05 in all 14 AML patients (AML_1 to AML_14) and 4 CD34+ healthy control cells confirmed inv(16) cluster (in ellipse).

The hierarchal clustering analysis was done with the R package Pvclust [10] using the correlation as a distance measure and Ward’s method. Only positions of autosomes (i.e. excluding mitochondria and sex chromosome calls) with coverage at least 10 and available for all samples (n = 2 010 310) were included in the analysis. From the clinical and molecular characteristics, only *CBFB*-*MYH11* patients (samples 1 and 2) clustered together. In Figure 1A, clustering of all CpGs is shown (for separate clustering of CpGs inside or outside CpG islands see the Additional file 1: Figure S1A and S1B). None of the other molecular abnormalities formed clusters and we did not observe any effect of clinical status of AML (*de novo*, therapy-related, or AML with myelodysplasia-related changes). One of the inv(16) sample was derived from BM (sample 1) while the other (sample 2) from PB. Importantly, this had no effect on their clustering consistency. We investigated this uniformity between the results from BM and PB in more details using 454 pyrosequencing in a larger number of patients (see below).

Clustering of gene expression data for 8 884 genes with detection P value ≤ 0.05 in all 14 AML patients and 4 CD34+ healthy control cells confirmed the inv(16) cluster (Figure 1B). Consistent with the DNA methylation data, no other characteristics formed clusters. The selection of these 14 AML patients was done randomly throughout various clinical subgroups (de novo, secondary after MDS, secondary after breast cancer, AML with dysplasia) with the aim to find common DNA methylation changes among diverse AML subtypes. Therefore, we cannot make any general conclusions with regard to the existence/non-existence of specific DNA methylation/gene expression profiles other than those demontrated in inv(16) AML, which were sufficiently verified in a validation cohort (see below).

### Hypomethylation signature of inv(16) AML patients

We extracted genomic regions being uniquely differentially methylated in *CBFB*-*MYH11* patients. There was a clear tendency towards hypomethylation with 125 out of 182 regions (69%) displaying lower DNA methylation levels compared to the healthy donors’ CD34+ pool of cells. All *CBFB*-*MYH11* DMRs were uploaded to GREAT [11] and enrichment for genes previously described as upregulated in inv(16) AML patients was observed (reported in ref. [8], ID: VALK_AML_CLUSTER_9; Additional file 2: Table S1). This enrichment set comprised 10 genomic regions assigned to 6 genes – *MN1*, *SPARC*, *ST18*, *DHRS3*, *FAM171A* and *BAHCC1* (see Additional file 3: Table S2). *MN1*, *SPARC*, *ST18*, *FAM171A* and *DHRS3* were chosen for hypomethylation validation by 454 bisulfite pyrosequencing. As we were primarily interested in hypomethylation associated with overexpression, *BAHCC1* was excluded from the methylation/expression validation, because its expression levels were undetectable in AML as well as in healthy donors’ samples according to microarray expression data. For *MN1* and *SPARC*, two regions per gene were studied.

**Figure 2:**
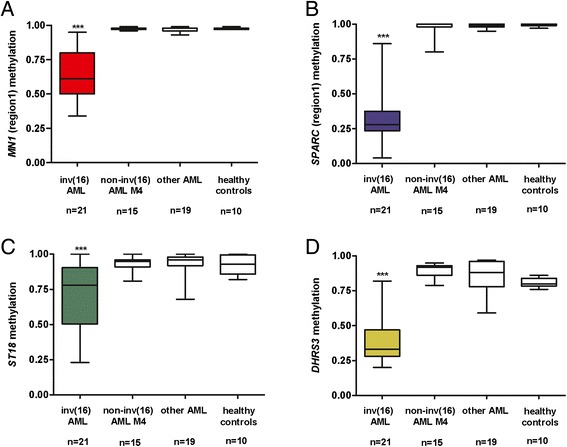
**Hypomethylation signature in inv(16) AML.** Hypomethylation of *MN1* **(A)**, *SPARC* **(B)**, *ST18* **(C)** and *DHRS3* **(D)** regulatory regions in inv(16) patients compared to AML M4 without inv(16), other AML subtypes and healthy controls; asterisks correspond to statistically significant changes of expression in inv(16) patients versus other groups, ***P < 0.0001.

In summary, altogether 21 inv(16) AML, 15 non-inv(16) AML M4, 19 other AML (1 AML M0, 3 AML M1, 6 AML M2, 3 AML M3, 3 AML M5a, 2 AML M5b, 1 AML M6) and 10 healthy controls were examined. DNA methylation of individual regions (corresponding to individual amplicons) was expressed as an average DNA methylation of all CpGs in that particular region. Average levels of DNA methylation in assigned regulatory regions of *MN1*, *SPARC*, *ST18* and *DHRS3* were significantly lower for inv(16) versus non-inv(16) AML M4, other AML subtypes and healthy controls (P < 0.0001) (see Figure 2). Sequencing of *FAM171A* failed twice in all samples probably due to the low complexity of the amplicon (because of the long stretches of identical bases – homopolymers) introduced after bisulfite conversion and *FAM171A* was therefore excluded from further analysis. For *MN1*, both of the studied regions displayed lower levels of methylation (for *MN1*_region_2 hypomethylation graph see Additional file 4: Figure S2A). For *SPARC*, the second studied region (*SPARC*_region_2) had low DNA methylation levels in inv(16) as well as in other AML and healthy donors (see Additional file 4: Figure S2B). Therefore only methylation of region 1 has a potential impact on *SPARC* expression. The 454-pyrosequencing results point to the site-specific *CBFB*-*MYH11* hypomethylation signature of genomic regions assigned to *MN1*, *SPARC*, *ST18* and *DHRS3*.

As we used either PB or BM as a starting material, we investigated whether there is a concordance between DNA methylation results from PB and BM in 10 AML patients with both materials at diagnosis available. We found high correlation between PB and BM results for all of the studied regions (R = 0.96).

**Figure 3:**
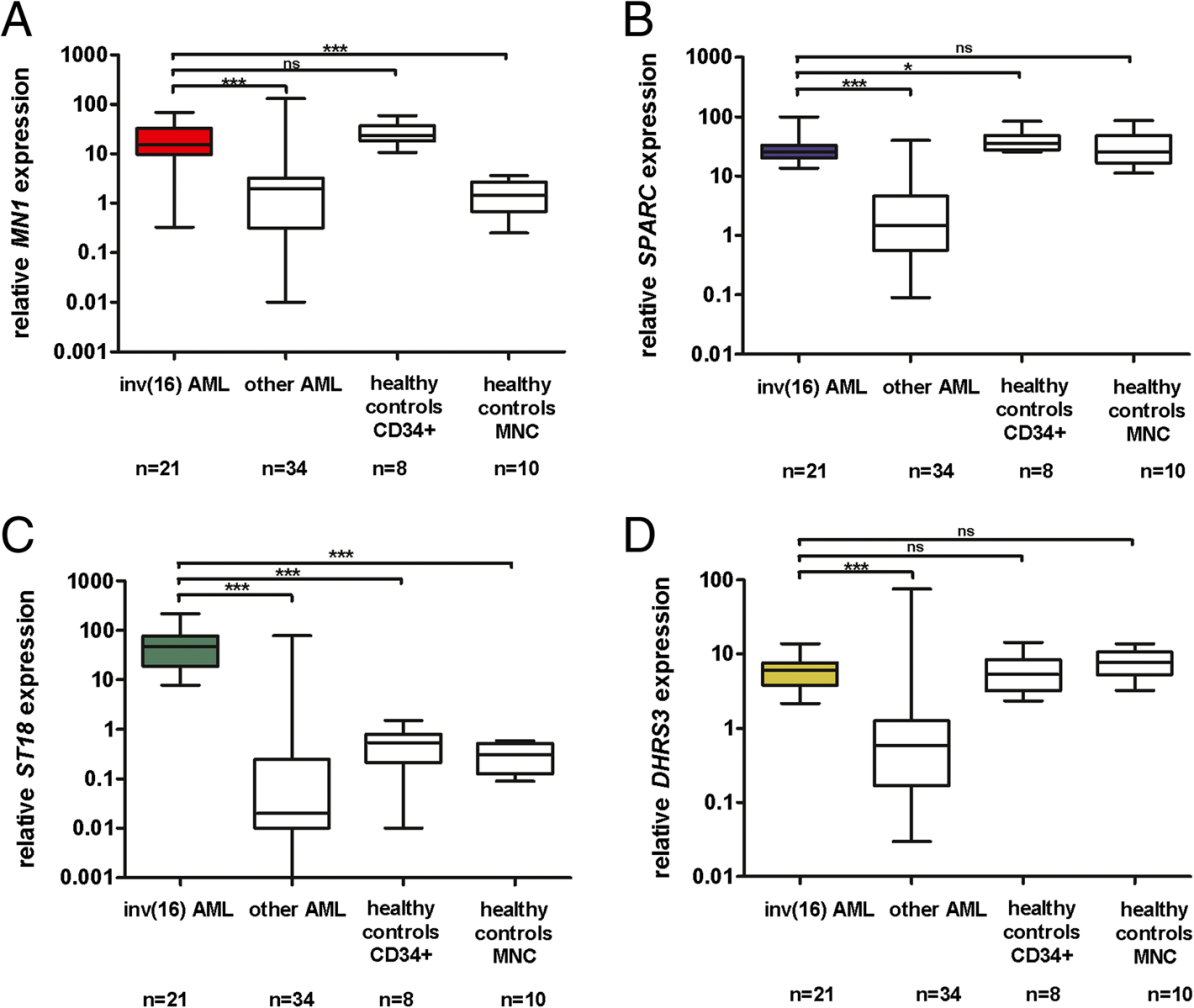
**Expression of hypomethylated genes in inv(16) AML measured by TaqMan gene expression assays.** Relative expression value of *MN1* **(A)**, *SPARC* **(B)**, *ST18* **(C)** and *DHRS3* **(D)** in inv(16) AML patients versus other AML subtypes and healthy control cells; MNC – mononuclear cells; asterisks correspond to statistically significant changes of expression, ***P < 0.0001, *P < 0.05, ns – not significant.

Further, we evaluated expression levels of *MN1*, *SPARC*, *ST18* and *DHRS3* by TaqMan gene expression assays in all samples already examined by 454 pyrosequencing. As can be seen in Figure 3, inv(16) patients had higher average levels of expression for all 4 genes in comparison to other non-inv(16) AML samples, but only in *ST18* when compared to healthy donors.

**Figure 4:**
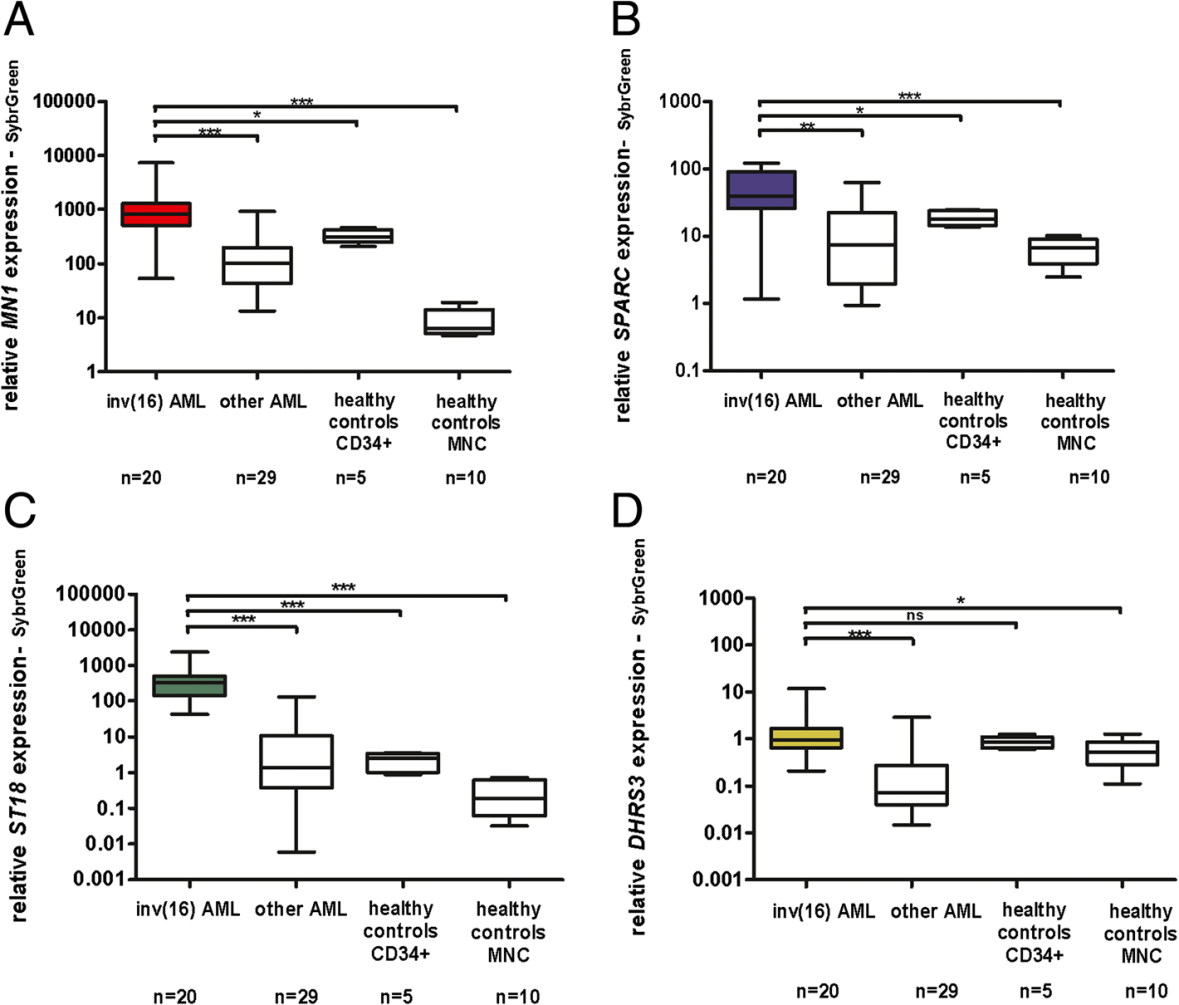
**Expression of hypomethylated genes in inv(16) AML measured by SybrGreen RQ-PCR.** Relative expression value of *MN1* **(A)**, *SPARC* **(B)**, *ST18* **(C)** and *DHRS3* **(D)** in inv(16) AML patients versus other AML subtypes and healthy control cells; MNC – mononuclear cells; asterisks correspond to statistically significant changes of expression, ***P < 0.0001, *P < 0.05, **P < 0.01, ns – not significant.

Accordingly, *ST18* was the most overexpressed gene in inv(16) AML compared to CD34+ cells of healthy controls in the microarray expression data. However, expression for all 3 remaining genes – *MN1*, *SPARC*, and *DHRS3* – was also upregulated in inv(16) AML (approximately 3-times higher than in CD34+ cells of healthy controls). Due to this inconsistency, we decided to re-measure gene expression by the use of SybrGreen RQ-PCR. This approach gave us results more similar to those observed by Illumina expression microarrays. As seen in Figure 4, differences in the gene expression levels between inv(16) AML and healthy controls increased significantly for *MN1* and *SPARC*. For *DHRS3*, the ratio of expression between inv (16) and healthy donors changed only slightly, and for *ST18* remained the same. One possible explanation of this discrepancy is the genomic location of primers. SybrGreen RQ-PCR primers for *MN1* and *SPARC* are located within the same exons as are the probes on the Illumina expression microarray, while TaqMan primers for *MN1* and *SPARC* have different, exon-exon locations. On the contrary, both types of primers (TaqMan and SybrGreen) for *ST18* and *DHRS3* have exon-exon locations. We checked SybrGreen RQ-PCR products specificity by melting analysis in all samples and no unspecificity was detected. It seems that in this particular case the selection of PCR detection system may influence results in a considerable manner. In general, this issue deserves further exploration.

In summary, *ST18* is the only overexpressed gene in inv(16) compared to other AML subtypes and healthy donors, irrespectively of the RQ-PCR system used.

### Correlation of methylation data with expression

For each transcription start site (TSS), a window was defined from 5 kb up- to 1 kb downstream of that TSS. Targeted regions overlapping this window were associated with the gene of the given TSS. GENCODE *v14* gene annotation was used for assigning DNA methylation of target regions to corresponding genes. Expression and methylation data were then correlated using Spearman rank tests. The small sample size did not allow for filtering based on P values and thus an ad hoc measure was employed, requiring strong anti-correlation (R ≤ −0.7), and a change of methylation between control and at least one AML sample 2-fold or greater (with either the control or at least one of the AMLs having methylation ratio 0.3 or greater). These strict filtering parameters resulted in a list of 163 genomic regions assigned to 130 unique genes (see Additional file 5: Table S3). Among these genes, we chose *PBX3* and *GFI1* to validate the association between DNA methylation and gene expression changes observed in this cohort of 14 AML patients.

### PBX3 and *GFI1* differential methylation is involved in their expression regulation

The DMRs associated with *PBX3* are located downstream (−160 to −1451 bp) of annotated CpG island (for exact location see Additional file 5: Table S3). We focused on a DMR encompassing a *TAF1* binding site (chr9:128 510 974–128 511 259 according to GRCh37/hg19). *TAF1* encodes the largest subunit of TFIID and this subunit binds to core promoter sequences encompassing TSSs.

**Figure 5:**
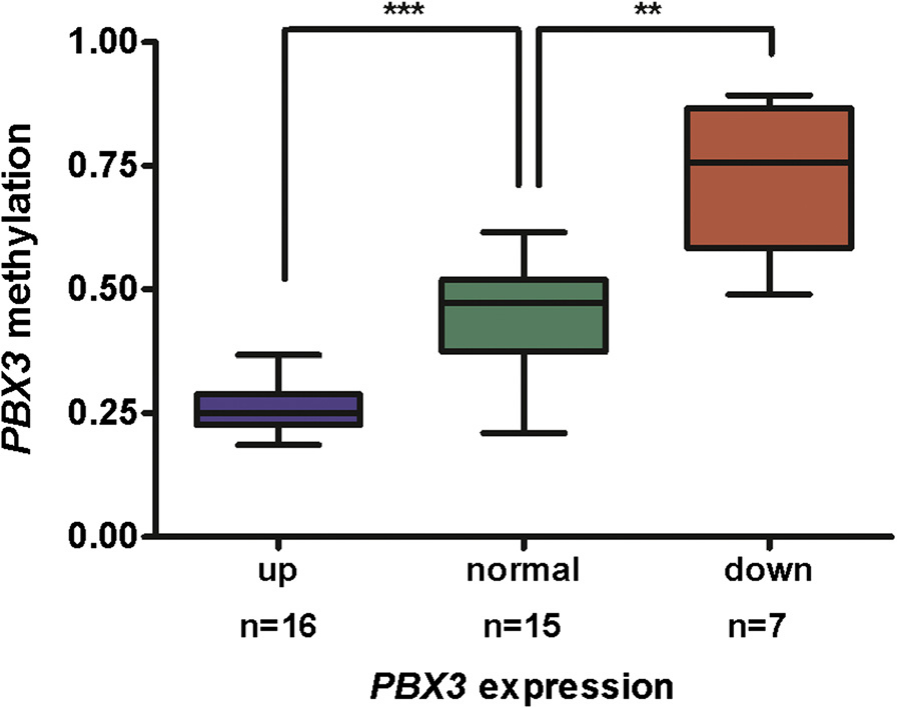
**PBX3 expression levels associated with DNA methylation of its downstream located regulatory region.** DNA methylation levels in AML patients with upregulated (up), downregulated (down) and normal levels of *PBX3* expression demonstrating the impact of *PBX3* methylation on expression; upregulation and downregulation was defined as a change in expression of at least one order of magnitude as well as 2-fold from healthy donors’ average *PBX3* expression; asterisks correspond to statistically significant changes of methylation, ***P < 0.0001, **P = 0.002.

We measured the expression of *PBX3* mRNA in 123 AML diagnostic samples and 15 healthy controls (for a graph see Additional file 6: Figure S3). 24% of AML had down- and 22% upregulated *PBX3* expression (with a minimum change in the expression of more than 2-fold and at the same time of more than one order of magnitude from healthy donors’ *PBX3* average expression). 454 pyrosequencing established the role of DNA methylation in down- and upregulation of *PBX3* in 30 AML patients at diagnosis (all of them with blast count ≥ 60%; Figure 5). Elevated levels of *PBX3* were connected with hypomethylation of a regulatory region (median methylation level 0.25, range 0.15 – 0.36; P < 0.0001), whereas decreased levels of expression with hypermethylation (median methylation level 0.51, range 0.31 – 0.98; P = 0.002). Control samples of healthy donors and AML samples with normal *PBX3* expression displayed intermediate levels of methylation (median 0.35, range 0.19 – 0.51). These results demonstrate the link between *PBX3* expression and DNA methylation levels of the *PBX3* regulatory region located downstream of the *PBX3* annotated CpG island.

Regarding *GFI1*, we examined a 409 bp long region within the *GFI1* promoter (chr1:92 952 229–92 952 637). Average DNA methylation levels of healthy controls (n = 14) fluctuated around 3.86%. Very similar to that, DNA methylation levels of AML within normal gene expression range were 4.54%. Only a minority of AML samples (3/64) showed decreased *GFI1* expression levels. However, all 3 *GFI1*-downregulated patients displayed higher methylation levels – 22.8%, 28.9% and 19%, respectively. On the contrary, upregulation of *GFI1* was not connected with any significant changes of DNA methylation levels, probably due to the fact that normal DNA methylation levels were already low, therefore any further decrease would not have a functional role.

### *PBX3* expression levels and their impact on prognosis of AML patients

Because *PBX3* is one of the four genes, whose common expression signature was described as having an impact on overall survival (OS) in AML patients [12], we decided to evaluate its expression levels in terms of OS and relapse-free survival (RFS). Only patients receiving standard curative therapy and those who did not die during the first induction were included in this analysis. Altogether 40 AML patients were assessed, 21/40 had low and 19/40 had high expression levels. Low and high expression levels were defined as a change in expression of at least one order of magnitude as well as 2-fold from healthy donors’ average *PBX3* expression.

**Figure 6:**
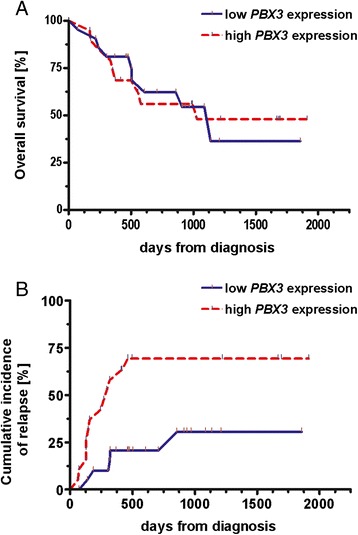
**Impact of** ***PBX3*** **expression levels on prognosis. (A)** Kaplan–Meier curves of overall survival (OS) in AML with low versus high expression of *PBX3* displaying no significant difference between these two groups of AML patients; **(B)** Cumulative incidence of relapse showing higher relapse rates in *PBX3*-overexpressing AML patients (P = 0.004).

We did not observe any effect of *PBX3* expression levels on OS in AML patients (Figure 6A), however we found higher relapse rates in AML patients with overexpressed *PBX3* (Figure 6B, P = 0.004).

We also performed multivariate analysis for both OS and RFS. Firstly, we tested the following parameters by univariate analysis: age, white blood count (WBC), complete remission after induction therapy (CR after induction), good/intermediate/poor prognosis according to cytogenetics, *PBX3* expression and *FLT3*-ITD status. For OS, only CR after induction was statistically significant in both univariate and multivariate analyses (P < 0.001). For RFS, parameters significant in univariate testing (WBC, CR after induction, *FLT3*-ITD status and *PBX3* expression) were evaluated by Cox regression. Only CR after induction and *PBX3* expression retained statistical significance (P = 0.002 and P = 0.028, respectively) in multivariate testing.

Furthermore, we evaluated whether there is an association between *PBX3* expression levels and presence of *MLL*-PTD or belonging to prognostically adverse AML subgroup. No such correlation was found, on the other hand there was no *PBX3*-overexpressing patient among AML patients within the prognostically favourable subgroup (0/6 cases from prognostically favourable versus 26/46 cases from prognostically intermediate or adverse subgroup overexpress *PBX3*, 0% versus 57% respectively, P = 0.02). Leukemic transformation mediated by MLL-fusion proteins has been suggested to be dependent on the presence of *PBX3* expression [9]. In our cohort, all three *MLL*-translocated patients, two with *MLLT3*-*MLL* (*MLL*-*AF9*) and one with *MLL*-*MLLT1* (*MLL*-*ENL*), had upregulated *PBX3* expression, but the low number of *MLL* translocations limited statistical testing of this association.

## Discussion

Here, we report a *CBFB*-*MYH11*, i.e. inv(16), specific hypomethylation that may play a role in upregulation of some previously described inv(16) overexpressed genes [8]. We analysed DNA methylation and expression data of *MN1*, *SPARC*, *ST18* and *DHRS3* in 55 AML patients and 10 healthy controls. Lower methylation levels of these genes in inv(16) patients versus other AML and healthy donors were confirmed. When measured by TaqMan gene expression assays, inv(16)-specific overexpression of *MN1*, *SPARC*, *ST18* and *DHRS3* was found with respect to non-inv(16) AML, but only in *ST18* in comparison with healthy donors. This was inconsistent with the Illumina microarray expression data as well as previously published data [8]. Therefore, we re-measured the results using SybrGreen RQ-PCR and we obtained different values. In this RQ-PCR experiment, changes in expression levels (between inv(16) AML and healthy donors) were also significant for *MN1* and *SPARC*. We excluded both the role of PCR nonspecificity and DNA contamination. Interestingly, *MN1* and *SPARC* primers for SybrGreen RQ-PCR, and *MN1* and *SPARC* probes on the expression microarray, are localized within the same exons, while *MN1* and *SPARC* TaqMan probes have different, exon-exon localizations. We cannot claim that this is the only reason for the different results, this issue definitively needs deeper examination in future studies. It is of interest that the results obtained by SybrGreen RQ-PCR are in agreement with the publicly available data (GSE34823) of the study of Bletiere et al. [13].

Average gene expression levels of *MN1*, *SPARC* and *ST18* extracted from the above-mentioned dataset are higher in inv(16) AML in comparison with healthy donors’ bone marrow (4-times for *MN1* and *SPARC*, 7-times for *ST18*), and *DHRS3* expression is basically the same in both groups.

Further, we extracted the data from The Cancer Genome Atlas (TCGA, https://tcga-data.nci.nih.gov/tcga/) and they also confirmed a link between hypomethylation of *MN1* and *DHRS3* regulatory regions and their overexpression in inv(16) AML when compared with AML samples with normal karyotype (healthy controls data were not available). Regulatory regions corresponding to remaining genes lacked information of their methylation status in TCGA due to the absence of appropriate CpGs in HumanMethylation450 BeadChip used at TCGA study [3]. This supports the profitability of studying DNA methylation using targeted bisulfite sequencing, which provides more complex coverage than microarray based techniques.

The hypomethylation pattern that we discovered in inv(16) AML patients is remarkable also with respect to the very recently published data of Mandoli and colleagues [14]. For the first time, their study revealed the involvement of *CBFB*-*MYH11* not only in repression but as well in transcriptional activation. The direct involvement of *CBFB*-*MYH11* in overexpression of *MN1*, *ST18* and *SPARC* is supported with 2-fold downregulation of these genes upon *CBFB*-*MYH11* knockdown as reported in their work. However, none of the 1874 high-confidence *CBFB*-*MYH11* binding sites [14] overlaps with any of the hypomethylated regions reported here. *DHRS3* was among the genes upregulated upon *CBFB*-*MYH11* knockdown, which is in agreement with its disputable upregulation in inv(16) AML. There were great differences in localization of hypomethylated regions with respect to TSSs of individual genes. With regard to *MN1* and *SPARC*, the hypomethylation was located not far from their TSSs (for location see Additional file 3: Table S2), which makes the assumption of their role in the expression of these genes more straightforward. Moreover, *MN1* and *SPARC* potential regulatory regions overlap regions of active chromatin (enhancers) in mobilized CD34+ cells as observed in the EpiGenome Browser (http://epigenomegateway.wustl.edu/browser) suggesting a role of these regions in transcription regulation. On the contrary, differentially hypomethylated sites assigned by GREAT to *ST18* and *DHRS3* are placed much farther from their TSSs, specifically approximately 275 kilobases (kb) downstream for *ST18* and 277 kb upstream for *DHRS3. ST18* and *DHRS3* assigned regulatory regions are placed within chromatin marked with low transcription activity and enhancer, respectively (in mobilized CD34+ cells, data from EpiGenome Browser).

*MN1* expression levels have been shown to stratify prognosis of cytogenetically normal (CN) AML patients and its overexpression is connected with a poor outcome of CN-AML patients [15,16]. Nevertheless, inv(16) AML patients are generally associated with a good prognosis [17,18] in spite of their frequent *MN1* overexpression. Functional studies have proved that overexpression of *MN1* cooperates with inv(16) in developing AML in vivo and that neither inv(16) or *MN1* alone are capable of promoting leukemia [19]. According to our results it seems that hypomethylation is present uniquely in inv(16) AML patients in both *MN1* assigned regions (none of the other AML subtypes or healthy controls displayed *MN1* hypomethylation in our cohort). So it supports the theory that upregulation of some genes that might involve *MN1* is crucial for inv(16) leukemogenesis and hypomethylation may be therefore needed to ensure stable overexpression of critical genes. Apparently the mechanism of *MN1* upregulation is different in non-inv(16) AML patients or potentially hypomethylation of other regulatory areas located elsewhere may be involved.

Correlation between targeted DNA methylation and microarray expression data of 14 AML patients and a healthy controls’ CD34+ pool revealed *PBX3* differential methylation and gene expression. *PBX3* (pre-B-cell leukemia homeobox 3) is part of the three amino acid loop extension (TALE) family of transcription factors, which include the products of the Pbx and Meis genes and are capable of heterodimerization with the Hox proteins [20]. Recently, *PBX3* was reported to have a synergistic effect with *HOXA9* in transforming normal hematopoietic progenitor cells in vitro as well as in vivo [8]. Moreover, *PBX3* is one of the four genes (*HOXA6*, *HOXA9*, *PBX3* and *MEIS1*), whose common expression signature was shown to influence overall survival in CN-AML [12]. All evidence points to an important role of *PBX3* in leukemogenesis. This is the first report uncovering DNA methylation as a plausible regulator of *PBX3* expression. We found a strong negative correlation between levels of *PBX3* methylation and expression in 8 healthy donors’ samples and 30 AML patients at diagnosis (P < 0.0001 and P = 0.002 for upregulation and downregulation, respectively). Localization of *PBX3* differential methylation overlaps *TAF1* binding site according to ENCODE ChIP-Seq data from UCSC genome browser. TAFs (TBP-associated factors) create a stable complex with *TBP* (TATA-binding protein) and *RNAPII* to form a preinitiation complex, so we may assume that DNA methylation status of *TAF1* binding site can directly influence the accessibility of DNA for transcription enzymes. The probability of transcription initiation is possibly dependent on whether the DNA methylation is low with high expression rates or DNA methylation is high with decreased expression or finally intermediate DNA methylation corresponding to in-between expression levels. As *PBX3* has a CpG island (CGI) overlapping its TSS, we also looked at its methylation status. Based on targeted methylation data, there was no methylation present either in AML or healthy controls. Therefore, methylation status of downstream located control element rather than CGI methylation is most likely crucial for *PBX3* expression. Further we focused on potential prognostic significance of *PBX3* expression in terms of overall survival (OS) and incidence of relapse. High *PBX3* expression levels were not related to different OS compared to AML patients with low *PBX3* expression, however relapse rates were significantly higher in *PBX3*-overexpressing patients by both univariate and multivariate testing. This suggests more aggressive phenotype/course of disease of these patients, which is not reflected in the OS probably due to the early and effective treatment of relapses – often followed by bone marrow transplantation. We also showed that *PBX3* overexpression did not occur in AML patients within cytogenetically favourable subgroup.

We validated the methylation/expression correlation stated in the Additional file 5: Table S3 also for *GFI1*. Moreover, the observed correlations are further supported by the presence of genes, for which the role of DNA methylation is already published such as *MPO* [21], *CEBPα*, *DAPK1*, *IRF8* and *PRDX8* [22,23].

## Findings

We found a new hypomethylation signature specific for inv(16) AML patients that may be responsible for overexpression of some genes that are crucial for inv(16) pathogenesis. *MN1* gene is likely to be a key gene involved in the pathogenesis of inv(16) AML and hypomethylation in the regulatory region near its TSS in inv(16) AML patients was confirmed, even on the basis of publicly available data from TCGA. Furthermore, we explored new regulatory region for *PBX3* and association of its methylation with *PBX3* expression. Therefore, targeted bisulfite sequencing represents a convenient approach in terms of genome coverage and informativeness with a great potential to reveal new regulatory regions of genes involved in leukemic transformation.

## Materials and methods

### Patients

For targeted bisulfite sequencing, 14 AML patients at diagnosis (see Table 1) and pooled CD34+ cells from 4 healthy donors were sequenced. Genes selected based on targeted bisulfite sequencing results were examined using 454 bisulfite pyrosequencing in a larger cohort of AML patients (their characteristics are given in Additional file 7: Table S4). Informed consent was obtained from all patients and healthy blood donors enrolled in the study. The study was approved by the IHBT Institutional Ethics Committee according to the Helsinki Declaration.

### Sample preparation

Mononuclear cells (MNC) from peripheral blood (PB) or bone marrow (BM) of the AML patients at diagnosis were separated by Ficoll gradient centrifugation (Histopaque, Sigma-Aldrich, Steinheim, Germany). CD34+ cells were harvested from buffy coats of healthy blood donors using MicroBead kits (Miltenyi Biotec GmbH, Bergish Gladbach, Germany). The CD34+ pool was created by mixing of 4 individual healthy blood donors’ separated cells (all of them men aged 42 to 58 years old, median age 45.5). DNA and RNA were extracted using AllPrep DNA/RNA Mini Kit (Qiagen, Hilden, Germany). Bisulfite conversion was performed from 1 μg of DNA by EpiTect Bisulfite Kit (Qiagen) and eluted into 40 μl of EB buffer. cDNA was prepared using M-MLV RT (Moloney Murine Leukemia Virus Reverse Transcriptase, Promega, Madison, WI, USA).

### Targeted bisulfite sequencing

Preparation of targeted bisulfite libraries started with 3 μg of genomic DNA and was carried out using SureSelect^XT^ Human Methyl-Seq kit (Agilent, Agilent Technologies, Santa Clara, CA, USA) according to the manufacturer’s instructions. Libraries were multiplexed into 4 pools and each pool was sequenced on 2 HiSeq™2000 (Illumina, San Diego, CA, USA) lanes using 105 bp paired-end sequencing reads with average coverage of 83 – ranging from 46 to 131.

### 454 bisulfite pyrosequencing

Bisulfite-treated (BS) DNA was subjected to 2-round PCR. In the 1st round of PCR, loci-specific primers with M13 universal tails were used to amplify regions of interest. Subsequently, primers specific to M13 universal tails now tailed with 454 -specific sequencing primers and a unique barcode sequence (MID) were applied to the 2nd PCR. Loci-specific primers were designed with Methyl Primer Express v1.0 software (Applied Biosystems Inc. Foster City, CA, USA; see Additional file 8: Table S5 for primer sequences). HotStarTaq DNA polymerase (Qiagen) and manufacturer’s recommended PCR reaction conditions were used for amplification. 2 μl of BS DNA was added to the 1st PCR and 1 μl of 100× diluted 1st round PCR product was subjected to the 2nd PCR. PCR cycling conditions were as follows: 1st round PCR – initial denaturation (15 min at 95°C), followed by 35 cycles of denaturation (30s at 94°C), annealing (30s at Ta °C, Ta – annealing temperature, see Additional file 8: Table S5) and extension (1 min at 72°C), final extension (10 min at 72°C); 2nd round of PCR - initial denaturation (15 min at 95°C), followed by 26 cycles of denaturation (30s at 94°C), annealing (30s at 60°C) and extension (1 min at 72°C), final extension (5 min at 72°C). All amplicons after 2nd round of PCR (up to 288 for one run) were purified using Agencourt AMPure^XP^ magnetic beads (Beckman Coulter, Fullerton, CA, USA) and Biomek® FXP Laboratory Automation Workstation (Beckman Coulter). Precise concentration of amplicons were determined using Quant-iT™ PicoGreen dsDNA Assay Kit (Life Technologies, Carlsbad, CA, USA) and amplicons were equimolarly pooled to obtain amplicon library with 10^9^ fragments/μl concentration. Subsequent procedures were carried out according to 454 amplicon sequencing manuals (454 Life Sciences, Roche Applied Science, Branford, CT, USA) on the GS Junior sequencer (Roche). An average overall coverage of 225 reads was observed (ranging from 53 to 659 for individual amplicons).

### mRNA microarray profiling

Gene expression profiles from 14 AML patients and 4 CD34+ cells of healthy controls were generated by HumanHT-12 v4 Expression BeadChip Kit (Illumina). The chip scanning was done with a BeadStation 500 instrument (Illumina).

### Quantitative real-time PCR (RQ-PCR)

The expression levels of selected genes were assessed with TaqMan Gene Expression Assays (Life Technologies) – see Additional file 9: Table S6 for individual Assay IDs. *GAPDH* was utilized as a house-keeping gene. Amplification was carried out with TaqMan Universal Master Mix II (Life Technologies) and recommended cycling conditions. SybrGreen RQ-PCR was performed using QuantiTect SYBR Green PCR Kit (Qiagen) and pre-designed KiCqStart® SYBR® Green primers (Sigma-Aldrich). Each sample was run in duplicates on a StepOne instrument (Life Technologies).

### Molecular genetics

The presence of internal tandem duplication (ITD) in the juxtamembrane (JM) and tyrosine kinase 1 (TKD1) domains (exons 12–14) of *FLT3* gene and the presence of *CBFB*-*MYH11* fusion transcript at diagnosis was detected as described previously [24]. Further, we examined mutations in *NPM1* [25], *CEBPA* [26] and *DNMT3A* [27] and intragenic *MLL* abnormalities such as partial tandem duplications (*MLL*-PTD) by direct sequencing [28,29].

### Cytogenetics

For cytogenetic analyses and fluorescence in situ hybridization (FISH) the samples of bone marrow were cultivated for 24 hrs in medium RPMI 1640 with 10% of fetal calf serum. Twenty G-banded Wright-Giemsa stained mitoses, if available, were evaluated. The karyotypes were described following ISCN 2013 nomenclature. For precise identification of chromosomal aberrations, FISH with locus specific DNA probes (Vysis, Downers Grove, IL, USA) and multicolor FISH with color kit probes and ISIS computer analysis (both from Metasysteme, Altlusheim, Germany) were used.

### Data processing and statistics

Data from targeted bisulfite sequencing were processed and evaluated using freely available programs: (i) FastQC [30] (quality control of reads), (ii) Trimmomatic [31] (removal of adapters/primer-dimers and bases with low-quality scores), (iii) Bismark [32] (methylation-aware alignment of reads to the reference genome and computation of methylation ratios). Differentially methylated target regions were assigned to genes using the on-line annotation tool GREAT [11]. Quantile normalization and subtraction of background was applied to the raw microarray expression data in BeadStudio Data Analysis Software (Illumina). Raw data from 454 pyrosequencing were processed using a filter template to relax the stringency of the original valley filter (kindly provided by Dr. Esteban Czwan, Roche). This step was necessary due to the lower complexity of bisulfite treated DNA containing long stretches of homopolymers. The filter template is available on-line (Additional file 10) and its usage is described in Additional file 11. Data from 454 pyrosequencing were aligned to a reference in GS Amplicon Variant Analyzer (AVA) (Roche) software and DNA methylation levels were assessed using the web-based software BISMA [33].

Kaplan–Meier curves and two-sided log-rank test were used to estimate the overall survival and to compare differences between survival curves. The relations between qualitative parameters were compared in contingency tables using Fisher’s exact test. For analyses of quantitative data, medians were detected and non-parametric two-tailed Mann–Whitney tests were performed. All these tests were conducted at a level of significance of 0.05 using GraphPad Prism4 software (GraphPad Software, San Diego, CA, USA). Cox regression analysis. was performed applying the SPSS statistical software (SPSS Inc., Chicago, IL, USA).

## Additional files

Additional file 1: Figure S1. Inv(16) methylation clusters inside and outside of CGIs. Hierarchal DNA methylation clustering of CpGs inside (A) and outside (B) of CGIs using the correlation as a distance measure and Ward’s method (AML_1 to AML_14 – AML patients; CD34_pool – healthy control’s CD34+ pool) indicating *CBFB*-*MYH11* methylation cluster (in ellipse) consistently with clustering of all CpGs shown in Figure 1. Additional file 2: Table S1. Enrichments for inv(16) uniquely differentially methylated regions (DMRs) as observed in GREAT. Enrichment terms observed after uploading of uniquely inv(16) DMRs to on-line annotation tool GREAT [11]. Additional file 3: Table S2. Inv(16) enriched regions. Genomic regions that were enriched for and associated with genes being previously described as upregulated in inv(16) AML. Additional file 4: Figure S2. Region_2 methylation levels of *MN1* and *SPARC* locus. (A) *MN1*_region 2 hypomethylation in inv(16) patients compared to AML M4 without inv(16), other AML subtypes and healthy controls; (B) *SPARC*_region 2 methylation levels are the same when compared AML M4 without inv(16), other AML subtypes and healthy controls. Additional file 5: Table S3. Genes with strong negative correlation between TSS adjacent DNA methylation levels and their expression. Genes with strong anti-correlation (R ≤ −0.7) between DNA methylation and expression, and with change of methylation between control and at least one AML sample 2-fold or greater (with either the control or at least one of the AMLs having methylation ratio 0.3 or greater); 163 genomic regions assigned to 130 unique genes. Additional file 6: Figure S3. Comparison of *PBX3* expression levels. *PBX3* relative gene expression levels in AML patients at diagnosis versus healthy controls’ samples. Additional file 7: Table S4. Samples used for inv(16) hypomethylation validation. Characteristics of AML patients examined by 454 bisulfite pyrosequencing to validate inv(16) hypomethylation of selected loci; BM – bone marrow, PB – peripheral blood. Additional file 8: Table S5. 454 bisulfite primers and annealing temperatures Primer sequences and annealing temperatures used to amplify regions of interest. Additional file 9: Table S6. TaqMan gene expression assays IDs. IDs of TaqMan probes used to measure gene expression of selected genes. Additional file 10:
**Filter template.** This template was used for 454 raw sequencing data reprocessing as described in Additional file 10. Additional file 11:
**454 pyrosequencing – raw data acquisition.** Description of amplicon filter template usage that allowed to obtain more reads from 454 bisulfite sequencing.
