# Supplementary figures and images for: CBFB-MYH11 hypomethylation signature and PBX3 differential methylation revealed by targeted bisulfite sequencing in patients with acute myeloid leukemia
Source: J Hematol Oncol. 2014 Sep 30;7:66. doi: 10.1186/s13045-014-0066-4 (PMC4197269; doi:10.1186/s13045-014-0066-4)

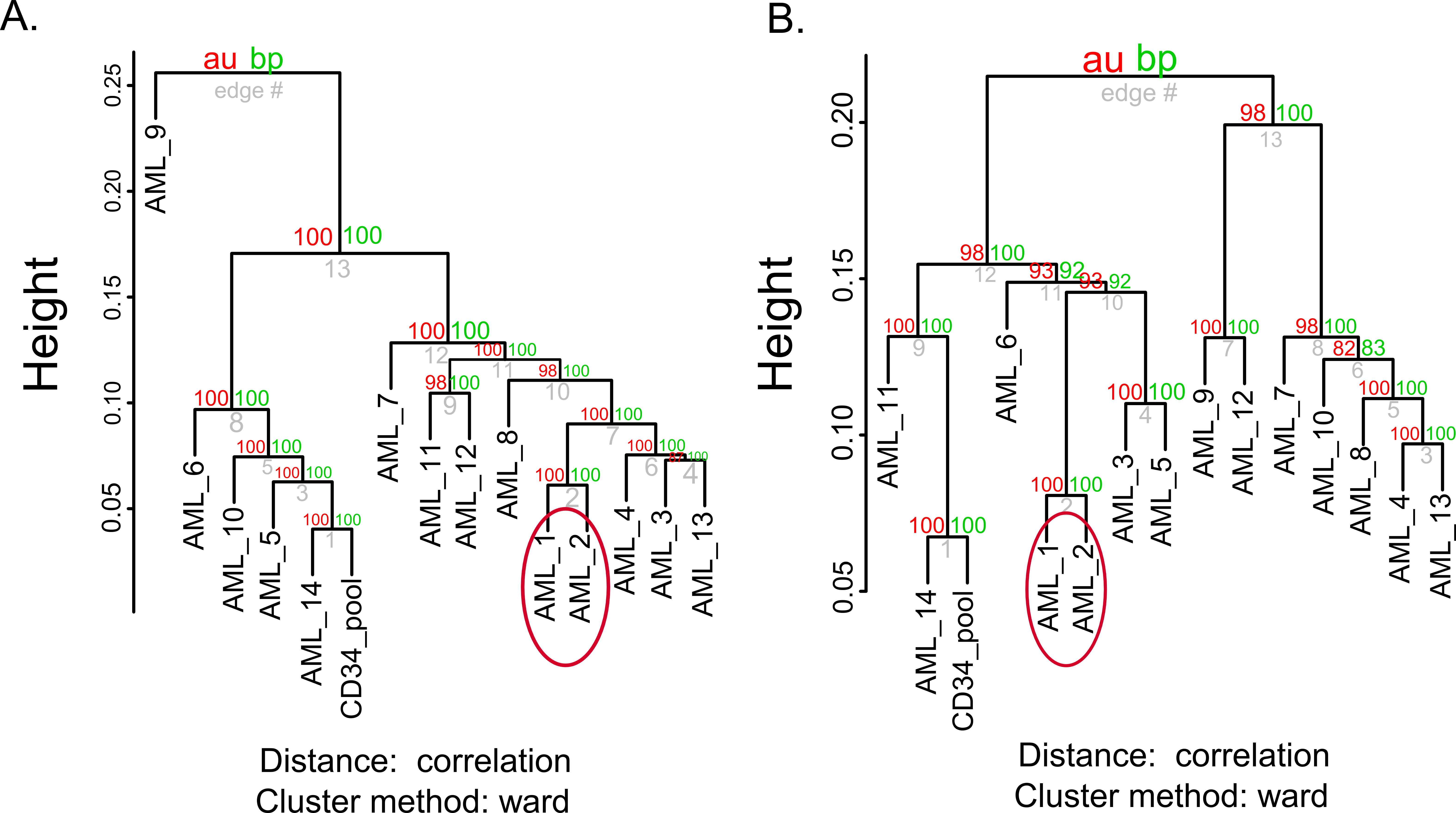

Supplement: Additional file 1: Figure S1. — Inv(16) methylation clusters inside and outside of CGIs. Hierarchal DNA methylation clustering of CpGs inside (A) and outside (B) of CGIs using the correlation as a distance measure and Ward’s method (AML_1 to AML_14 – AML patients; CD34_pool – healthy control’s CD34+ pool) indicating CBFB-MYH11 methylation cluster (in ellipse) consistently with clustering of all CpGs shown in Figure 1. [file 13045_2014_66_MOESM1_ESM.png]

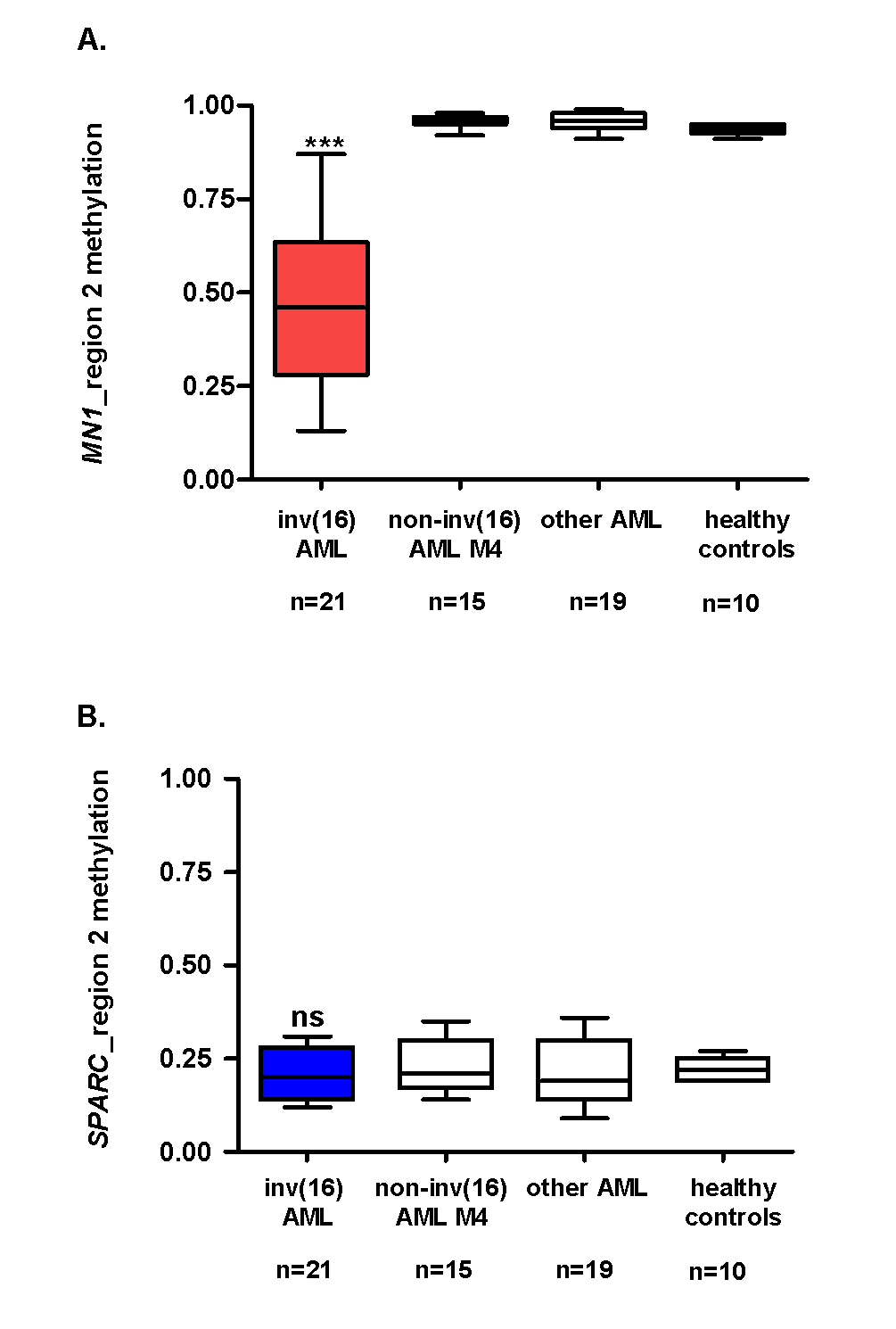

Supplement: Additional file 4: Figure S2. — Region_2 methylation levels of MN1 and SPARC locus. (A) MN1_region 2 hypomethylation in inv(16) patients compared to AML M4 without inv(16), other AML subtypes and healthy controls; (B) SPARC_region 2 methylation levels are the same when compared AML M4 without inv(16), other AML subtypes and healthy controls. [file 13045_2014_66_MOESM4_ESM.tiff]

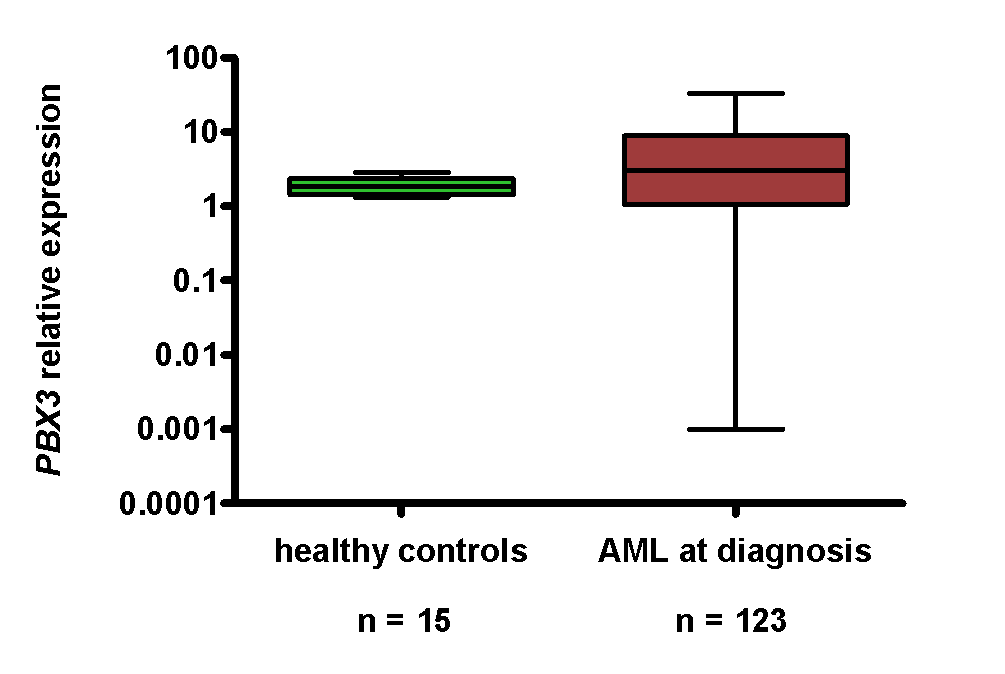

Supplement: Additional file 6: Figure S3. — Comparison of PBX3 expression levels. PBX3 relative gene expression levels in AML patients at diagnosis versus healthy controls’ samples. [file 13045_2014_66_MOESM6_ESM.tiff]
