# Supplementary material for: CBFB-MYH11 hypomethylation signature and PBX3 differential methylation revealed by targeted bisulfite sequencing in patients with acute myeloid leukemia
Source: J Hematol Oncol. 2014 Sep 30;7:66. doi: 10.1186/s13045-014-0066-4 (PMC4197269; doi:10.1186/s13045-014-0066-4)
Supplement: Additional file 11: — 454 pyrosequencing – raw data acquisition. Description of amplicon filter template usage that allowed to obtain more reads from 454 bisulfite sequencing. [file 13045_2014_66_MOESM11_ESM.docx]

**Additional file 10**

**454 pyrosequencing – raw data acquisition**

For obtaining more reads, we used an amplicon filter template (available on-line ***bisulfite_amplicon_v2.7_02.xml***) allowing more valleys and scanning fewer flows. This approach was used to re-run the quality filters with a relaxed pipeline on the already full processed data.

To analyse raw data after 454 sequencing run follow these steps:

1. Copy the file (bisulfite_amplicon_v2.7_02.xml) to any location in your Junior attendant PC (for example to /home/adminrig).
2. Open a terminal window (right-click on the desktop and select ‘Open Terminal’) and type the following command:

“runAnalysisFilter--pipe=/home/adminrig/bisulfite_amplicon_v2.7.xml D_fullProcessing”

D_fullProcessing is the D_fullProcessing folder of the run in question (please note that you may need to provide the full path to the folder, for example, /data/2013_03_05/R_2013_xx_xxxxx/D_fullProcessing)

1. Running this command will take approximately 15-20 minutes, depending on the amount of data. As final outcome, a new D_ folder will be created inside the R_ folder. This new folder called D_bisulfite_amplicon_v2.7 will contain a new SFF file with the new set of reads.
